# Long noncoding RNA CCAT1 functions as a ceRNA to antagonize the effect of miR-410 on the down-regulation of ITPKB in human HCT-116 and HCT-8 cells

**DOI:** 10.18632/oncotarget.21612

**Published:** 2017-10-07

**Authors:** Bo Li, Chong Shi, Jingming Zhao, Bai Li

**Affiliations:** ^1^ Department of Gastrointestinal Colorectal and Anal Surgery, China-Japan Union Hospital of Jilin University, Changchun, P.R. China; ^2^ Department of Anorectal Surgery, The Afflicted Hospital to Changchun University of Chinese Medicine, Changchun, P.R. China; ^3^ Department of Colorectal and Anal Surgery, The First Affiliated Hospital of Jilin University, Changchun, P.R. China

**Keywords:** colon cancer, miRNA-410, *CCAT1*, *ITPKB*

## Abstract

Colorectal cancer is one of the most common malignancies, which has seriously affected people's health. Abnormal expression of long non-coding RNAs and microRNAs are closely related to the process of occurrence, development, invasion and metastasis of colorectal cancer. However, the effect of lnc *CCAT1* on human HCT-116/HCT-8 cells and its potential mechanism were investigated. In present study, differential expression of *CCAT1*, miR-410 and *ITPKB* were detected in colon cancer tissues and adjacent parts. Then the prediction programs were applied to predict the target genes of miR-410. The complementary bindings of miR-410 with lnc *CCAT1* and *ITPKB* were assessed by luciferase assays. The interaction between LncRNA *CCAT1* and miR-410 was analyzed. In addition, the mRNA and protein of *ITPKB* and apoptosis factors were examined in cells after miR-410 overexpression or silencing. Meanwhile, MTT and flow cytometer were used to detect the cells proliferation and apoptosis level. Results showed that *CCAT1* and miR-410 were up-regulated in colon cancer tissues, but *ITPKB* was down-regulated. Lnc *CCAT1* and *ITPKB* were predicted to be the targets of miR-410 and the prediction were verified by luciferase assays. The expression of lnc *CCAT1* and *ITPKB* were inhibited by miR-410 in human HCT-116/HCT-8 cells. Meanwhile, lnc *CCAT1* could lead to a decrease of miR-410. Furthermore, miR-410 overexpression could promote cell proliferation and reduce apoptosis. In summary, these data demonstrated that miR-410 could promote cell proliferation and reduce apoptosis by inhibiting *ITPKB* expression and the expression of lnc *CCAT1* antagonized the effect of miR-410.

## INTRODUCTION

Colorectal cancer (CRC) is one of the most common malignant tumors of the digestive tract caused serious harm to human life and health [1]. The morbidity and mortality of colorectal cancer are higher due to a variety of factors including colon polyps, ulcerative colitis, lifestyle, diet, age, obesity, environment and genes interaction [2, 3]. Thus, the effective methods are urgently needed for the CRC patients’ treatment. However, its pathogenesis has not been fully elucidated.

Long non-coding RNAs (lnc RNAs) are formed by RNApol II transcription with a length of more than 200nt non-coding RNA molecules and have no protein coding function due to the lack of effective open reading frame (ORF) [4, 5]. *CCAT1* was first found to be up-regulated in colorectal cancer [6]. Previous studies have found that *CCAT1* could be activated by *c-Myc* and played a regulatory role in the development, progression, metastasis and invasion of colorectal cancer [7–10]. Furthermore, hepatocellular carcinoma [11, 12], gastric cancer [9], gallbladder cancer [13], breast cancer [14] cells proliferation and migration levels were up-regulated which were confirmed to be related to the abnormal expression of *CCAT1*. However, the regulation mechanism of *CCAT1* in colon cancer is still not clear.

MicroRNAs (miRNAs) are highly conserved RNAs participate in a series of life process by regulating the expression of genes resulting in mRNAs degradation or post-transcriptional inhibition [15]. Studies have showed that miRNAs are widely involved in the occurrence, development, prognosis and recurrence of colorectal cancer [16–20]. Among the reported miRNAs, miR-410 has be proved to function as a tumor suppressor in human glioma though regulating *MET* [21]. ITPKB, a member of 3-kinases family, is associated with calcium signaling pathway. It may play a vital role in immune disorders, Alzheimer's disease, multiple sclerosis and malignant melanoma [22, 23]. Meanwhile, research has shown that *ITPKB* could be down-regulated by miR-375 in SCLC and promote cell growth in SCLC cell line [24]. However, little work has been reported on miR-410 and *ITPKB* function in colon cancer.

In the present study, we aimed to investigate the expression, function and interaction of miR-410, *CCAT1* and *ITPKB* in human HCT-116 cells to reveal the underlying mechanisms. Our findings demonstrated that miR-410 could inhibit cell proliferation and promote apoptosis by inhibiting *ITPKB* expression and the expression of *CCAT1* antagonized the effect of miR-410 on the down-regulation of its target *ITPKB* in human HCT-116 cells. Which laid the foundation for the deeply study of colon cancer.

## RESULTS

### Patient statistics

A total of 30 colon cancer patients were included in this study. The surgeries were performed from January 2010 to December 2011. The colon cancer tissues and adjacent parts were collected and stored at -80°C. The proportion of male was 63.3% and the average age was 60.9 ± 12.1. All the patients were Han population in north of China.

### The genes expression in different colon cancer tissues

**Figure 1:**
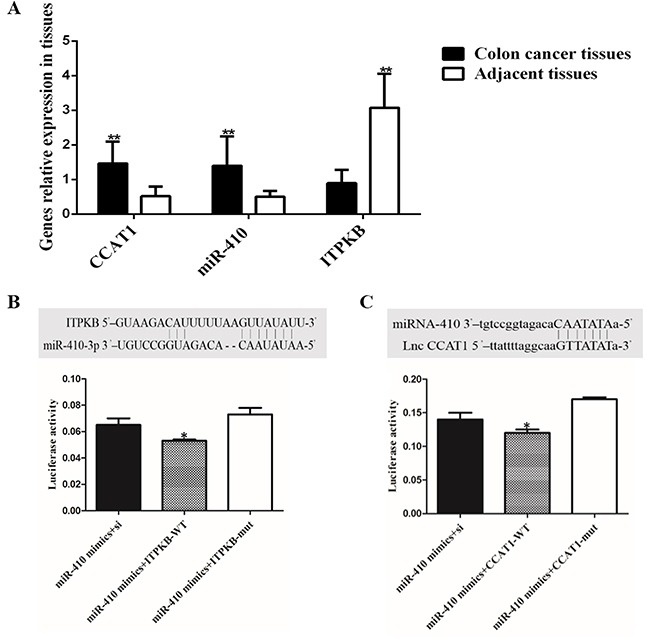
Relative expression of genes mRNA in colon cancer tissue and target genes of miR-410 validation **(A)** miR-410, *CCAT1* and *ITPKB* mRNA in colon cancer tissues and adjacent part; **(B)** Binding site and binding capacity between miR-410 and *ITPKB*; **(C)** Target site and luciferase activity were analyzed for relationship between miR-410 and *CCAT1*. ^*^p<0.05 was a significantly different; ^**^p<0.01, the difference was extremely significant.

Differences in expression of miR-410, *CCAT1* and *ITPKB* were detected in 30 different colon cancer tissues and adjacent parts using qPCR. As shown in Figure 1A, miR-410 and *CCAT1* expression levels were gradually up-regulated in colon cancer tissues (p<0.01), but the expression of *ITPKB* mRNA was significantly higher in adjacent parts (p<0.01) (Figure 1A).

### MiR-410 directly target *ITPKB* and *CCAT1*

The prediction programs (Target Scan, starBase v2.0 and miRGen) were used to identify potential binding sites with miR-410 in the 3′UTR and the target relationship was higher between the miR-410 and *ITPKB/*lnc *CCAT1* 3′UTR among the results. Besides, *ITPKB* and *CCAT1* had been generally proved to participate in cancer cell proliferation. In our previous research, miR-410 and *CCAT1* was markedly up-regulated and *ITPKB* expression level was gradually reduced in colon cancer tissues and adjacent parts. As shown in Figure 1B-1C, miR-410 could closely bind to the target sites in the 3′UTR of *ITPKB/*lnc *CCAT1*. Compared with the control group, luciferase activity was significantly lower in group (miR-410 mimics + ITPKB-WT) and group (miR-410 mimics + CCAT1-WT). Luciferase results showed that miR-410 had a high binding ability with *ITPKB/*lnc *CCAT1* 3′UTR (Figure 1B-1C) (p<0.05).

### MiR-410 down-regulated *ITPKB* and the interaction between miR-410 and lnc *CCAT1*

**Figure 2:**
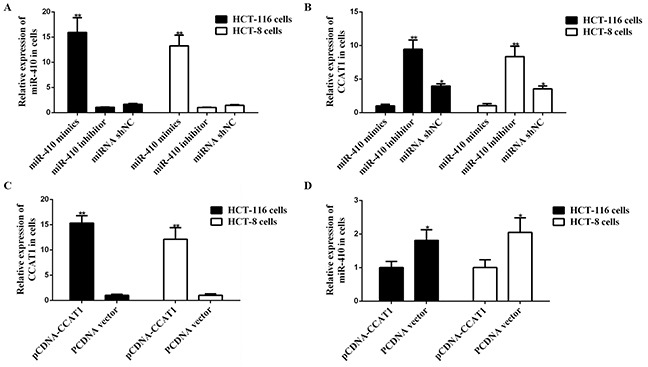
Interaction between miR-410 and lnc *CCAT1* **(A-B)** miR-410 mimics, inhibitor and miR-shNC were transfected into HCT-116/HCT-8 cells, then *CCAT1* were analyzed by qPCR. **(C-D)** Effect of *CCAT1* on miR-410 were analyzed in HCT-116/HCT-8 cells. ^*^p<0.05 was a significantly different; ^**^p <0.01, the difference was extremely significant.

**Figure 3:**
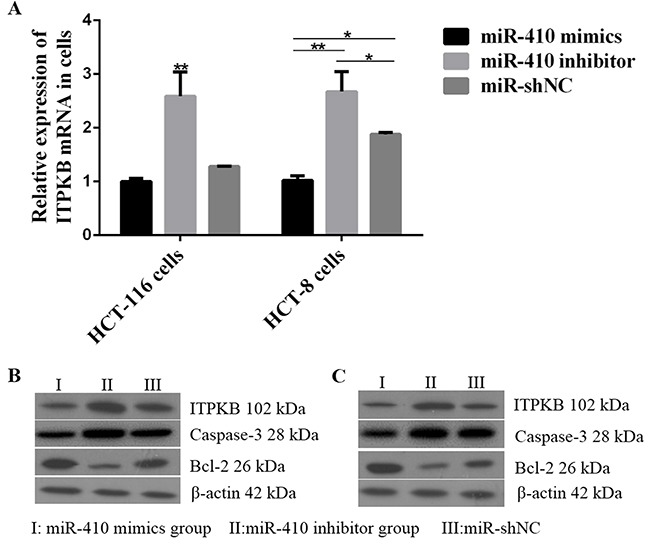
Effects of miR-410 on *ITPKB* and apoptosis factors in HCT-116/HCT-8 cells **(A)** *ITPKB* mRNA expression levels detected by qPCR after miR-410 mimics or miR-410 inhibitor or miR-shNC transfecting into HCT-116/HCT-8 cells. **(B)** ITPKB and apoptosis factors proteins were detected in HCT-116 cells. **(C)** miR-410 mimics or miR-410 inhibitor or miR-shNC transfected into HCT-8 cells, then ITPKB and apoptosis factors proteins were detected by western blot. ^*^p<0.05 was a significantly different; ^**^p<0.01, the difference was extremely significant.

As shown in Figure 2A-2B, lnc *CCAT1* was significant down-regulated in miR-410 mimics transfecting cells, and a higher expression was found in cells transfected with miR-410 inhibitor. On the other hand, lnc *CCAT1* was widely expressed in HCT-116 cells and HCT-8 cells transfecting with pCDNA-CCAT1 (Figure 2C). Compared with the control group, lnc *CCAT1* overexpression could down-regulate miR-410 (Figure 2D). Therefore, there was an interaction between miR-410 and lnc *CCAT1*. Besides, *ITPKB* mRNA and protein expressed in miR-410 inhibitor group was significantly increased, indicating that miR-410 could reduce the expression of *ITPKB* (p<0.01) (Figure 3).

### MiR-410 promote the human HCT-116/HCT-8 cells proliferation and inhibit cells apoptosis

**Figure 4:**
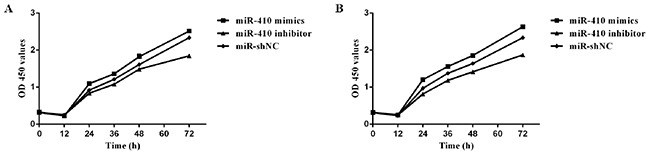
HCT-116/HCT-8 cell proliferation ability **(A)** HCT-116 cell growth rate counted after transfection. **(B)** Growth rate were detected in HCT-8 cells transfected miR-410 mimics, inhibitor and miR-shNC.

**Figure 5:**
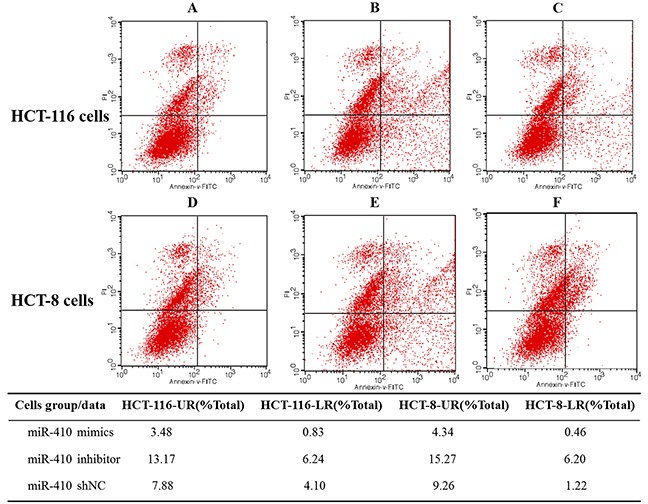
HCT-116/HCT-8 cell apoptosis rate **(A-C)** HCT-116 cells transfected miR-410 mimics, inhibitor and miR-shNC, then apoptosis rate was detected by flow cytometer. **(D-F)** HCT-8 cells apoptosis rate was detected after transfection. ^**^p<0.01, the difference was extremely significant.

The human HCT-116/HCT-8 cells proliferation and apoptosis were detected by MTT and flow cytometry after miR-410 transfection. Results showed that miR-410 mimics group induced a significant increase on the growth rate of human HCT-116/HCT-8 cells (p<0.01) (Figure 4). After 48 h transfection, the apoptosis results showed that the apoptosis rates were 4.31% (miR-410 mimics group), 19.41% (miR-410 inhibitor group) and 11.98% (miR-shNC) in HCT-116 cells. Meanwhile, the apoptosis rates were 4.8% (miR-410 mimics), 21.47% (miR-410 inhibitor) and 10.48% (miR-shNC) in HCT-8 cells. Suggesting that miR-410 could inhibit both HCT-116 and HCT-8 cells apoptosis (Figure 5).

### The caspase-3 and Bcl-2 expression levels detection

We further detected the apoptosis related protein caspase-3 and Bcl-2 after miR-410 overexpression in HCT-116 and HCT-8 cells. The results showed that caspase-3 level was higher in miR-410 inhibitor group compared with miR-410 shNC group. However, Bcl-2 protein level showed an opposite trend. In other words, *ITPKB* up-regulation caused an increase of caspase-3 and a decrease of Bcl-2 (Figure 3B).

## DISCUSSION

Nowadays, tumor resection and concurrent chemo radiotherapy are the main means for the treatment of CRC, however, it has a poor prognosis. Lnc RNAs and miRNAs have been verified to participate in cancer development and progression by regulating the cell proliferation, differentiation and apoptosis [25–32]. Besides, the potential role played by these molecules in the pathogenesis of CRC attracts more and more attention, however, the current mechanism research is still not comprehensive. Our study aims to investigate the interaction among lnc *CCAT1*, miR-410 and its target gene *ITPKB* in human HCT-116/HCT-8 cells proliferation and apoptosis.

So far, a variety of miRNAs have been shown to be involved in the pathogenesis of colon cancer, such as miR-34a [33], miR-506 [34], miR-675 [35] and miR-200 [36]. In previous study, miR-410 has be proved to function as a tumor suppressor in human glioma by regulating *MET*. Thus, miR-410 was chose to conduct further research. The prediction programs (Target Scan, starBase v2.0 and miRGen) were used to identify potential binding sites and the target relationship between miR-410 and *ITPKB/*lnc *CCAT1* was verified using luciferase assays. Luciferase assays showed that miR-410 could target *ITPKB/*lnc *CCAT1* 3′UTR. The qPCR and western blot results on the tissues confirmed this relationship. Meanwhile, miR-410 mimics could promote the human HCT-116/HCT-8 cells proliferation and attenuate apoptosis. In addition, the caspase-3 expression level was reduced and Bcl-2 was significant increase in miR-410 mimics group.

Lnc *CCAT1* has been involving in the development, progression, metastasis and invasion of colorectal cancer [8, 10]. Emerging evidence have confirmed that lnc RNAs might function as a competing endogenous RNA (ceRNA) or a molecular sponge in modulating miRNAs. Meanwhile, studies indicated that lnc *CCAT1* could negatively regulate the expression of miRNA-218-5p and let-7 in gallbladder cancer [13] and hepatocellular carcinoma [11]. In addition, there were less research on lnc *CCAT1* in colon cancer. In this study, we predicted that the miR-410 target sites were in the 3′UTR of lnc *CCAT1*. Dual-luciferase reporter assay confirmed the interaction between miR-410 and lnc *CCAT1* which was similar to previous studies [37]. Lnc *CCAT1* overexpression could improve the ITPKB level. This relationship was detected on the tissue using qPCR and western blot.

In conclusion, the interaction among miR-410, *CCAT1* and *ITPKB* was detected in human HCT-116/HCT-8 cells. MiR-410 could directly target lnc *CCAT1* and *ITPKB*. Meanwhile, miR-410 mimics could promote the human HCT-116/HCT-8 cells proliferation and attenuate apoptosis. In addition, the caspase-3 expression level was reduced and Bcl-2 was significant increase in miR-410 mimics group. Simultaneously, lnc *CCAT1* functioned as a ceRNA to antagonize the effect of miR-410 on the down-regulation of *ITPKB*. These findings might provide a basis for the treatment of colon cancer.

## MATERIALS AND METHODS

### Tissue samples

The 30 CRC tissues and adjacent tissues were collected from the Department of Colorectal and Anal Surgery in Third Affiliated Hospital of Jilin University. The study was approved by the Ethics Committee of Third Affiliated Hospital of Jilin University. Tissue samples were stored in liquid nitrogen.

### Cell culture and transfection

The prediction programs (Target Scan, starBase v2.0 and miRGen) were applied to predict the target genes of miR-410. The human HCT-116/HCT-8 cell lines were purchased from Shanghai Institute for Biological Sciences (Shanghai, China). All cells were cultivated in DMEM medium (GIBCO, USA) with 10 % FBS (HyClone, USA), 100 units/ml penicillin and 100 mg/ml streptomycin at 37°C in incubator containing 5% CO_2_. The miR-410 mimics, miR-410 inhibitor, shNC, WT-vectors (*CCAT1-WT*, *ITPKB-WT*), mut-vectors (*CCAT1-mut*, *ITPKB-mut*), pCDNA-*CCAT1* and PCDNA3.1 vector were synthesized and purchased from RiboBio Company (Guangzhou, China). The HCT-116/HCT-8 cells transfection and co-transfection were performed using FuGENE HD Transfection Reagent (Promage, USA) according to the manufacturer's instructions.

### Real-time PCR and western blot detection

Total RNA was isolated using Trizol reagent and cDNAs were synthesized by a RT-PCR Kit (Takara, Japan). Specific primers were designed for RT-PCR and qPCR reaction (Table 1). The conditions and procedures were referred to the instructions. The proteins were extracted with RIPA buffer (Boster, China) and the BCA Protein Assay Kit (Boster, China) was used to detect protein concentration referring to the instructions. Proteins were isolated by SDS-PAGE and transferred to PVDF membrane. The membrane was blocked with 5% skim milk powder and then incubated with rabbit anti-ITPKB antibody, 1:200 ((Bioss, China), rabbit anti-Caspase-3 antibody, 1:300 (Bioss, China), rabbit anti-Bcl-2 antibody, 1:300 (Bioss, China) and mouse anti-β-actin antibody, 1:6000 (Bioss, China). The proteins bandings were detected with ECL Western Blotting Substrate (Invitrogen, USA).

**Table 1:** Primer sequences of qPCR

Symbol	Primer	Primer Sequence (5′–3′)
hsa-miR-410-3p	RT-Primer	CTCAACTGGTGTCGTGGAGTCGGCAATTCAGTTG
	F-Primer	AGACAGGCCA
	R-Primer	ACACTCCAGCTGGGAATATAACACAGATG
		CAGTGCAGGGTCCGAGGT
U6	RT-Primer	AACGCTTCACGAATTTGCGT
	F-Primer	CTCGCTTCGGCAGCACA
	R-Primer	AACGCTTCACGAATTTGCGT
ITPKB	F-Primer	TTAAAGCCATCTCGTCCCTAC
	R-Primer	GCCCAAAGCTCCATAAACAAC
CCAT1	F-Primer	CATTGGGAAAGGTGCCGAGA
	R-Primer	ACGCTTAGCCATACAGAGCC
β-actin	F-Primer	CTCCATCCTGGCCTCGCTGT
	R-Primer	GCTGTCACCTTCACCGTTCC

### Luciferase assays

The *ITPKB/CCAT1* wild type (WT), *ITPKB/CCAT1* mutant type (mut) and si were respectively co-transfected with mi-410 mimics into HCT-116/HCT-8 cells by FuGENE HD Transfection Reagent. Firefly and Renilla luciferase activities were measured using a dual-luciferase reporter gene assay system at 48 h after transfection.

### Cell proliferation and apoptosis assay

The HCT-116/HCT-8 cells were transfected with miR-410 mimic, miR-410 inhibitor and shNC. Cell cycle progression and levels of apoptosis were analyzed using Cell Cycle and Apoptosis Detection Kit (Beyotime Biotechnology, China) according to manufacturer's protocol.

### Statistical analysis

Data were reported as mean ± standard deviation (SD). The differences among groups were analyzed by one-way Analysis of Variance followed by Fisher's LSD test. Survival curves were plotted after the test. Statistical analysis results were performed using SPSS19.0 software for windows and a significant difference was considered with p<0.05.

## References

[1] Siegel RL, Miller KD, Jemal A (2015). Cancer statistics, 2015. CA Cancer J Clin.

[2] Siegel R, Desantis C, Jemal A (2014). Colorectal cancer statistics, 2014. CA Cancer J Clin.

[3] Vargas AJ, Thompson PA (2012). Diet and nutrient factors in colorectal cancer risk. Nutr Clin Pract.

[4] Wu Q, Kim YC, Lu J, Xuan Z, Chen J, Zheng Y, Zhou T, Zhang MQ, Wu CI, Wang SM (2008). Poly A- transcripts expressed in HeLa cells. PLoS One.

[5] Mercer TR, Dinger ME, Mattick JS (2009). Long non-coding RNAs: insights into functions. Nat Rev Genet.

[6] Nissan A, Stojadinovic A, Mitrani-Rosenbaum S, Halle D, Grinbaum R, Roistacher M, Bochem A, Dayanc BE, Ritter G, Gomceli I, Bostanci EB, Akoglu M, Chen YT (2012). Colon cancer associated transcript-1: a novel RNA expressed in malignant and pre-malignant human tissues. Int J Cancer.

[7] He X, Tan X, Wang X, Jin H, Liu L, Ma L, Yu H, Fan Z (2014). C-Myc-activated long noncoding RNA CCAT1 promotes colon cancer cell proliferation and invasion. Tumour Biol.

[8] McCleland ML, Mesh K, Lorenzana E, Chopra VS, Segal E, Watanabe C, Haley B, Mayba O, Yaylaoglu M, Gnad F, Firestein R (2016). CCAT1 is an enhancer-templated RNA that predicts BET sensitivity in colorectal cancer. J Clin Invest.

[9] Yang F, Xue X, Bi J, Zheng L, Zhi K, Gu Y, Fang G (2013). Long noncoding RNA CCAT1, which could be activated by c-Myc, promotes the progression of gastric carcinoma. J Cancer Res Clin Oncol.

[10] Ye Z, Zhou M, Tian B, Wu B, Li J (2015). Expression of lncRNA-CCAT1, E-cadherin and N-cadherin in colorectal cancer and its clinical significance. Int J Clin Exp Med.

[11] Deng L, Yang SB, Xu FF, Zhang JH (2015). Long noncoding RNA CCAT1 promotes hepatocellular carcinoma progression by functioning as let-7 sponge. J Exp Clin Cancer Res.

[12] Zhu H, Zhou X, Chang H, Li H, Liu F, Ma C, Lu J (2015). CCAT1 promotes hepatocellular carcinoma cell proliferation and invasion. Int J Clin Exp Pathol.

[13] Ma MZ, Chu BF, Zhang Y, Weng MZ, Qin YY, Gong W, Quan ZW (2015). Long non-coding RNA CCAT1 promotes gallbladder cancer development via negative modulation of miRNA-218-5p. Cell Death Dis.

[14] Zhang XF, Liu T, Li Y, Li S (2015). Overexpression of long non-coding RNA CCAT1 is a novel biomarker of poor prognosis in patients with breast cancer. Int J Clin Exp Pathol.

[15] Leonardo TR, Schultheisz HL, Loring JF, Laurent LC (2012). The functions of microRNAs in pluripotency and reprogramming. Nat Cell Biol.

[16] Amirkhah R, Schmitz U, Linnebacher M, Wolkenhauer O, Farazmand A (2015). MicroRNA-mRNA interactions in colorectal cancer and their role in tumor progression. Genes, Chromosomes Cancer.

[17] Ghanbari R, Mosakhani N, Asadi J, Nouraee N, Mowla SJ, Yazdani Y, Mohamadkhani A, Poustchi H, Knuutila S, Malekzadeh R (2015). Downregulation of plasma MiR-142-3p and MiR-26a-5p in patients with colorectal carcinoma. Iran J Cancer Prev.

[18] Hur K (2015). MicroRNAs: promising biomarkers for diagnosis and therapeutic targets in human colorectal cancer metastasis. BMB Rep.

[19] Okayama H, Schetter AJ, Harris CC (2012). MicroRNAs and inflammation in the pathogenesis and progression of colon cancer. Dig Dis.

[20] Yazdani Y, Farazmandfar T, Azadeh H, Zekavatian Z (2016). The prognostic effect of PTEN expression status in colorectal cancer development and evaluation of factors affecting it: miR-21 and promoter methylation. J Biomed Sci.

[21] Chen L, Zhang J, Feng Y, Li R, Sun X, Du W, Piao X, Wang H, Yang D, Sun Y, Li X, Jiang T, Kang C (2012). MiR-410 regulates MET to influence the proliferation and invasion of glioma. Int J Biochem Cell Biol.

[22] Nalaskowski MM, Fliegert R, Ernst O, Brehm MA, Fanick W, Windhorst S, Lin H, Giehler S, Hein J, Lin YN, Mayr GW (2011). Human inositol 1,4,5-trisphosphate 3-kinase isoform B (IP3KB) is a nucleocytoplasmic shuttling protein specifically enriched at cortical actin filaments and at invaginations of the nuclear envelope. J Biol Chem.

[23] Tajouri L, Mellick AS, Tourtellotte A, Nagra RM, Griffiths LR (2005). An examination of MS candidate genes identified as differentially regulated in multiple sclerosis plaque tissue, using absolute and comparative real-time Q-PCR analysis. Brain Res Brain Res Protoc.

[24] Jin Y, Liu Y, Zhang J, Huang W, Jiang H, Hou Y, Xu C, Zhai C, Gao X, Wang S, Wu Y, Zhu H, Lu S (2015). The expression of miR-375 is associated with carcinogenesis in three subtypes of lung cancer. PLoS One.

[25] Cui M, Xiao Z, Wang Y, Zheng M, Song T, Cai X, Sun B, Ye L, Zhang X (2015). Long noncoding RNA HULC modulates abnormal lipid metabolism in hepatoma cells through an miR-9-mediated RXRA signaling pathway. Cancer Res.

[26] Herriges MJ, Swarr DT, Morley MP, Rathi KS, Peng T, Stewart KM, Morrisey EE (2014). Long noncoding RNAs are spatially correlated with transcription factors and regulate lung development. Genes Dev.

[27] Hu X, Feng Y, Zhang D, Zhao SD, Hu Z, Greshock J, Zhang Y, Yang L, Zhong X, Wang LP, Jean S, Li C, Huang Q (2014). A functional genomic approach identifies FAL1 as an oncogenic long noncoding RNA that associates with BMI1 and represses p21 expression in cancer. Cancer Cell.

[28] Li Z, Lei H, Luo M, Wang Y, Dong L, Ma Y, Liu C, Song W, Wang F, Zhang J, Shen J, Yu J (2015). DNA methylation downregulated mir-10b acts as a tumor suppressor in gastric cancer. Gastric Cancer.

[29] Shi Y, Wang Y, Luan W, Wang P, Tao T, Zhang J, Qian J, Liu N, You Y (2014). Long non-coding RNA H19 promotes glioma cell invasion by deriving miR-675. PLoS One.

[30] Wang HJ, Ruan HJ, He XJ, Ma YY, Jiang XT, Xia YJ, Ye ZY, Tao HQ (2010). MicroRNA-101 is down-regulated in gastric cancer and involved in cell migration and invasion. Eur J Cancer.

[31] Wang P, Liu YH, Yao YL, Li Z, Li ZQ, Ma J, Xue YX (2015). Long non-coding RNA CASC2 suppresses malignancy in human gliomas by miR-21. Cell Signal.

[32] Yuan JH, Yang F, Wang F, Ma JZ, Guo YJ, Tao QF, Liu F, Pan W, Wang TT, Zhou CC, Wang SB, Wang YZ, Yang Y (2014). A long noncoding RNA activated by TGF-beta promotes the invasion-metastasis cascade in hepatocellular carcinoma. Cancer Cell.

[33] Jiang H, Ge F, Hu B, Wu L, Yang H, Wang H (2017). rs35301225 polymorphism in miR-34a promotes development of human colon cancer by deregulation of 3′UTR in E2F1 in Chinese population. Cancer Cell Int.

[34] Zhou H, Lin C, Zhang Y, Zhang X, Zhang C, Zhang P, Xie X, Ren Z (2017). miR-506 enhances the sensitivity of human colorectal cancer cells to oxaliplatin by suppressing MDR1/P-gp expression. Cell proliferation.

[35] Chen S, Bu D, Ma Y, Zhu J, Chen G, Sun L, Zuo S, Li T, Pan Y, Wang X, Liu Y, Wang P (2017). H19 overexpression induces resistance to 1,25(OH)2D3 by targeting VDR through miR-675-5p in colon cancer cells. Neoplasia.

[36] Yang W, Ning N, Jin X (2017). The lncRNA H19 promotes cell proliferation by competitively binding to miR-200a and derepressing beta-catenin expression in colorectal cancer. Biomed Res Int.

[37] Wang ZH, Guo XQ, Zhang QS, Zhang JL, Duan YL, Li GF, Zheng DL (2016). Long non-coding RNA CCAT1 promotes glioma cell proliferation via inhibiting microRNA-410. Biochem Biophys Res Commun.

